# Relative stopping power measurements to aid in the design of anthropomorphic phantoms for proton radiotherapy

**DOI:** 10.1120/jacmp.v15i2.4523

**Published:** 2014-03-06

**Authors:** Ryan L. Grant, Paige A. Summers, James L. Neihart, Anthony P. Blatnica, Narayan Sahoo, Michael T Gillin, David S. Followill, Geoffrey S. Ibbott

**Affiliations:** ^1^ The Graduate School of Biomedical Sciences The University of Texas Health Science Center Houston TX; ^2^ Department of Radiation Physics The University of Texas M.D. Anderson Cancer Center Houston TX; ^3^ Office of Medical Physics and Radiation Safety Boston University Medical Center Boston MA USA

**Keywords:** quality assurance, proton therapy, stopping powers

## Abstract

The delivery of accurate proton dose for clinical trials requires that the appropriate conversion function from Hounsfield unit (HU) to relative linear stopping power (RLSP) be used in proton treatment planning systems (TPS). One way of verifying that the TPS is calculating the correct dose is an end‐to‐end test using an anthropomorphic phantom containing tissue‐equivalent materials and dosimeters. Many of the phantoms in use for such end‐to‐end tests were originally designed using tissue‐equivalent materials that had physical characteristics to match patient tissues when irradiated with megavoltage photon beams. The aim of this study was to measure the RLSP of materials used in the phantoms, as well as alternative materials to enable modifying phantoms for use at proton therapy centers. Samples of materials used and projected for use in the phantoms were measured and compared to the HU assigned by the treatment planning system. A percent difference in RLSP of 5% was used as the cutoff for materials deemed acceptable for use in proton therapy (i.e., proton equivalent). Until proper tissue‐substitute materials are identified and incorporated, institutions that conduct end‐to‐end tests with the phantoms are instructed to override the TPS with the measured stopping powers we provide. To date, the RLSPs of 18 materials have been measured using a water phantom and/or multilayer ion chamber (MLIC). Nine materials were identified as acceptable for use in anthropomorphic phantoms. Some of the failing tissue substitute materials are still used in the current phantoms. Further investigation for additional appropriate tissue substitute materials in proton beams is ongoing. Until all anthropomorphic phantoms are constructed of appropriate materials, a unique HU‐RLSP phantom has been developed to be used during site visits to verify the proton facility's treatment planning HU‐RLSP calibration curve.

PACS number: 87.53.Bn

## I. INTRODUCTION

The mission of the Radiological Physics Center (RPC) is to assure the National Cancer Institute (NCI) that institutions participating in NCI‐funded clinical trials deliver radiation doses that are clinically comparable and consistent with the requirements of the trials. One of the ways the RPC monitors institutions is with anthropomorphic phantoms.^(1)^ These phantoms were originally designed using tissue‐equivalent materials that had physical characteristics to match patient tissues when irradiated with megavoltage photon beams.^2^, ^3^ In addition, the materials chosen had similar ratios of CT number (or Hounsfield unit value) to relative electron density as the corresponding biological materials. This helped to ensure that the traditional conversion tables used by many treatment planning systems would render the appropriate relative electron density value on the basis of CT data for these materials and, thus, calculate the dose to the tissue correctly. Cost, machining ability, uniformity, and rigidity were additional criteria that governed the choice of materials.

Proton therapy is an attractive modality for radiation therapy, in large part because the steep dose gradient at the end of the particle's path allows the delivery of a high dose to the target while keeping the dose to structures on the distal side of the target low. This can be advantageous for many patients and body sites, and has stimulated increased interest in using proton therapy not only in the radiation oncology community, but also within the clinical trial setting.^(4)^ In contrast to the megavoltage dose calculation depending on the electron density of a material, proton radiation therapy relies on a material's stopping power to describe the energy loss of protons due to the interactions in matter and to calculate proton doses. As such, the CT calibration curve in a proton treatment planning system relates Hounsfield unit (HU) values to relative stopping power.^5^, ^6^ This generally differs from the electron density of materials used in a HU calibration curve for photon therapy. As proton therapy gains traction as a treatment option across the United States, efforts to develop anthropomorphic phantoms suitable for the quality assurance of proton treatments have been escalated. At the same time, interest in clinical trials including or comparing proton therapy with other modalities has increased, and the RPC has been tasked to develop auditing procedures for proton therapy facilities. To enable remote audits of treatment planning and dose delivery, the RPC has begun to modify several of its anthropomorphic phantoms with tissue‐substitute materials suitable for proton dose measurements (proton‐equivalent).

As an initial step in this phantom modification process, materials being considered for use in phantoms intended to evaluate proton therapy were measured to determine their relative linear stopping power (RLSP) value. Alternative phantom materials were also measured to replace those photon phantom materials for which the material's RLSP was not within 5% of the HU‐RLSP calibration curve.

## II. MATERIALS AND METHODS

**Figure 1:**
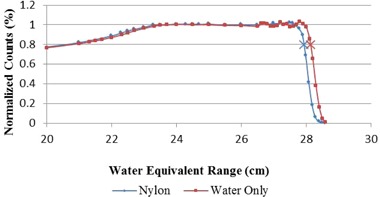
A portion of the depth dose curve in water with (circles) and without (squares) Nylon added to the surface. The curve is a cubic spline fit to the data. “x” indicates the location of the distal 80% dose of each curve.

The methodology modified from Schaffner and Pedroni and described by Moyers et al.^6^, ^7^ was used to compute the stopping power of the phantom material relative to water from the shift in the percent depth dose (PDD) curve, as indicated in Eq. (1) and shown in Fig. 1.

(1) $$RLSP=\frac{\Delta x}{t_{m}}$$

In Eq. (1), Δx is the change in the depth of the distal 80% PDD when the tissue‐substitute material is introduced into the beam, and $t_{m}$ is the thickness of the material. Both Δx and the material thickness are linear dimensions and must be expressed in consistent units. The uncertainty was calculated using the method from Moyers, seen in Eq. (2).^(6)^

(2) $$dRLSP=\frac{dR_{80,w}}{t_{m}}+\frac{dR_{80,m}}{t_{m}}+\frac{\left|\Delta x\right|dt_{m}}{t_{m}^{2}}$$

where $dt_{m}$ = uncertainty in material thickness, $dR_{80,w}$ = uncertainty in depth to distal 80% in water, and $dR_{80,m}$ = uncertainty in depth to distal 80% with material in place.

Two methods were used to measure Δx: depth dose scanning in a water phantom with a single ion chamber, and depth dose measurements made with a multilayer ion chamber (MLIC) (“Zebra”; IBA, Louvain‐la‐Neuve, Belgium). For measurements in an in‐house portable water phantom, a parallel plate chamber (Exradin P11; Standard Imaging, Middleton, WI) served as a scanning chamber, while an Exradin A12 Farmer‐type thimble chamber was placed at the edge of the field as a reference chamber. The Zebra MLIC consists of 180 parallel plate chambers separated by high‐density material chosen so that the average water‐equivalent thickness (WET) of the stack of chambers together with air is unity.^(8)^ The device is calibrated providing a WET within ±0.15 mm accuracy (standard deviation) at all depths. In this manner, a depth dose curve equivalent to that measured in a water phantom is formed from signals obtained from the MLIC chambers during the course of a single exposure. The distal 80% was chosen as the point of measurement. The WET uncertainty of the Zebra MLIC device may introduce a random uncertainty in the determination of the RLSP for each material that depends on the thickness of the material tested. The materials tested with the Zebra had thicknesses ranging from 4.3±0.05 cm to 7.9±0.05 cm in order to minimize the uncertainty to only 0.3%.

Measurements were made at the University of Texas MD Anderson Cancer Center Proton Therapy Center – Houston (PTC‐H) using a passively‐scattered beam delivered from a rotating gantry having a source‐to‐axis distance (SAD) of 270 cm. The treatment gantry was set at 270° and measurements were made with an SSD of 270 cm set to the entrance window of the water phantom. For convenience, the MLIC was positioned with its center at the isocenter. A reference PDD measurement (with no additional material in the path of the beam) was obtained with a 250 MeV beam having a range of 28.5 cm and a 5 cm spread‐out Bragg peak (SOBP). Each alternative phantom material was machined for uniform thickness. Multiple thickness measurements were taken with calipers for each phantom material to ensure uniform thickness to an uncertainty of ±0.05 mm (1 SD). The materials were affixed to the front of the entrance window of the water phantom or the MLIC on the central axis of the beam, and the PDD measurement was repeated for every material.

Each tissue‐substitute material was imaged in air with a GE LightSpeed RT16 CT scanner (GE, Waukesha, WI) at the PTC‐H using a 1.25 mm slice thickness. A conversion table of HU‐RLSP values had previously been determined using the stoichiometric method for use in proton therapy. The images were transferred to an Eclipse treatment planning system (TPS) (Varian Medical Systems, Palo Alto, CA), and the average Hounsfield unit value was determined in a 10 pixel region of interest. An RLSP for each tissue‐substitute material was assigned by the TPS using the clinical HU‐RLSP calibration curve. The thickness of each material studied, change of location of the distal 80% depth dose, and Eq. (1) were used to determine the measured RLSP values. Plotting the measured RLSP values against the planning system's calibration curve showed the discrepancies between the TPS‐assigned RLSP based on HU and the measured actual RLSP. The assigned RLSP for each material was derived from the calibration curve, and the percent difference between assigned and measured RLSP was calculated, as shown in Eq. (3).

(3) %Difference=MeasuredRLSP−AssignedRLSP(MeasuredRLSP+AssignedRLSP)2×100

## III. RESULTS

Figure 1 shows the percent depth dose curve with nylon in the beam compared to the curve obtained without additional material in the beam. This curve is representative of results that were generated for all of the tissue substitute materials. The RPC's pelvis phantom uses nylon to simulate the prostate. The “x” positioned on the distal end of the PDD curves marks the 80% depth dose point as calculated from a cubic spline interpolation.

**Figure 2:**
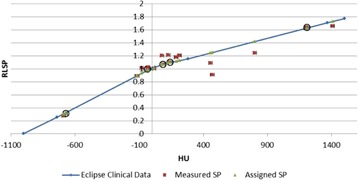
Materials with measured RLSP and assigned RLSP. Materials circled were included in the HU‐RLSP phantom, shown in Fig. 3. Error bars indicate uncertainty expressed as 1 standard deviation in the measured value.

**Figure 3:**
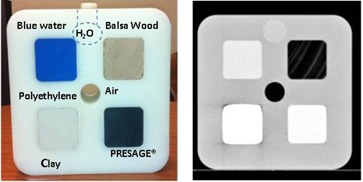
HU‐RLSP phantom and CT image of the phantom.

The RLSP values calculated according to the method of Schaffner and Pedroni, those assigned by the TPS, and the HU values transferred from the CT scanner to the TPS for each material tested to date are listed in Table 1. The percent difference is also listed in the final column for each material and is illustrated in Fig. 2. Based on the determination of the RLSPs for the materials listed in Table 1, a unique HU‐RLSP phantom was developed and is shown in Fig. 3.

**Table 1:** The materials evaluated for this study, the HU values transferred from the CT scanner to the TPS, the measured RLSP, the assigned RLSP, and the percent difference between the two are recorded. A double space marks the point at which the investigators changed from water phantom measurements to the Zebra MLIC

*Material*	*HU*	*Measured SP*	*Assigned SP*	*% Uncert.*	*% Diff.*
Acrylic (PMMA)	125	1.21	1.09	1.26%	10.3%
Wax	−80	1.01	0.93	0.83%	8.2%
Nylon	76.8	1.20	1.06	2.14%	12.2%
Polyethylene^a^	−34	1.00	0.98	1.91%	1.9%
PBT‐poly (Polybutylene	215	1.21	1.13	3.05%	6.7%
Teraphalate Polyester)					
High‐Impact Polystyrene^a^	−30	1.02	0.98	0.36%	4.1%
PVC (PolyVinyl Chloride)	800	1.25	1.42	0.37%	−13.0%
RMI Solid Water^a^	16	1.00	1.01	0.73%	−0.6%
Balsa Wood^a^	−672.3	0.31	0.33	1.07%	−4.2%
Pressed Cork	−690	0.28	0.31	2.83%	−10.7%
PRESAGE^a^ (LMG Formulation)	141.1	1.10	1.1	0.29%	0.0%
CIRS Bone^a^	1408	1.66	1.73	0.56%	−4.0%
Bone Meal	470	0.91	1.25	0.61%	−31.5%
Plaster of Paris	455	1.09	1.24	0.80%	−13.1%
Dense Iron Wood (Latin America Lignum)	188	1.18	1.12	0.33%	5.4%
Alderson Solid Water^a^ (Standard Imaging)	16	1.00	1.01	0.73%	−0.6%
Blue Water^a^	86	1.07	1.07	0.41%	−0.1%
Clay^a^	1207	1.64	1.63	0.28%	0.9%

^a^ Tissue‐substitute material considered to be suitable materials (within±5%)

## IV. DISCUSSION

The RPC anthropomorphic QA phantoms are used as one component of a credentialing program required of institutions wishing to participate in NCI‐sponsored clinical trials. They function as an end‐to‐end test, with treatment planning being a key component of the final analysis. The acceptance criteria for the phantoms require the delivered dose to be within a certain percentage of the planned absolute dose in low‐gradient regions and within a certain distance to agreement in high‐gradient regions. Many of the materials tested had measured RLSP values that were different by greater than 5% from the TPS‐predicted values. A 5% error in RLSP value for a material of 5 cm thickness can lead to a shift of 2.5 mm in the computed range of a proton beam. Incorrect RLSPs, therefore, can lead to proton range errors, resulting in dose deposited in the wrong locations. For institutions attempting to pass the RPC's credentialing tests, the use of incorrect RLSPs for the phantom materials can lead to errors that could mask errors in beam modeling or planning procedures.

The RLSP values reported here were measured with a proton beam energy of 250 MeV. There is a known dependence of RLSP on proton energy; however, this is more important at low‐proton energies. The choice of energy used here was based on the energies most often used at proton centers irradiating the RPC's anthropomorphic phantoms and the geometry of the phantoms.

When institutions receive the current RPC phantoms, they are instructed to override the RLSP values assigned by the TPS based on measured HU values and, instead, use RLSP or HU values provided by the RPC. This modification to the institution's TPS is sometimes confusing to the institution, resulting in errors. Unfortunately, it also bypasses a test of the accuracy of the clinic's HU‐RLSP calibration curve. The procedure to override the RLSPs is a temporary measure and is being followed while the RPC continues to test materials that can be evaluated for their proton equivalency. The RPC has modified its lung, brain, and liver phantoms to contain proton‐equivalent materials. It is expected that the RPC will construct new phantoms with proton‐equivalent materials for proton trial credentialing use only.

An HU‐RLSP phantom was developed by the RPC through its investigation of new proton equivalent tissue substitutes. The phantom consists of seven materials having RLSPs that agree with the RLSP of biological materials to within 5%, and which span HU values from 1140 (air) to +1139 (bone). It is proposed as a standard phantom that can be imaged and compared against a proton facility's clinical TPS HU‐RLSP curve. Since the materials in the phantom approximate very closely the human tissue HU‐RLSP curve, this phantom offers a unique QA tool without having to make extensive approximations and calculations required when nonproton‐equivalent materials are used to verify the planning system's HU‐RLSP curve. This phantom is now being used by the RPC during its dosimetry review visits to institutions wishing to use proton therapy in NCI‐funded clinical trials as a QA tool to verify consistency between proton therapy centers.

## V. CONCLUSIONS

Materials currently used or proposed for use in RPC phantoms were tested for proton therapy equivalency. Many tissue‐substitute materials considered acceptable for megavoltage photon beam QA were found to have greater than 5% difference in RLSP from the assigned value based on HU. However, several proton‐equivalent materials have been identified and now are being used in several of the RPC phantoms. Further investigation for additional appropriate proton‐equivalent tissue substitute materials is ongoing. Until all phantoms are constructed of proton‐equivalent materials, a unique HU‐RLSP phantom has been developed to be used during site visits to verify the proton facility's clinical treatment planning HU‐RLSP calibration curve.

## ACKNOWLEDGMENTS

Funding for this project was in part from Federal Share of program income earned by Massachusetts General Hospital on C06 CA059267 and grants CA10953 and CA81647.
